# A Multi-Objective Optimization Method for Cylindrical Surface Ultrasonic Array Parameters Based on BPNN and NSGA-II

**DOI:** 10.3390/s25216762

**Published:** 2025-11-05

**Authors:** Xin Zeng, Xueshen Cao, Jiaheng Zhao, Yuyu Dai, Chao Li, Hao Chen

**Affiliations:** 1 University of Chinese Academy of Sciences, Beijing 100049, China; zengxin@mail.ioa.ac.cn (X.Z.); chaoli@mail.ioa.ac.cn (C.L.); 2 Institute of Acoustics, Chinese Academy of Sciences, Beijing 100190, China; zhaojiaheng@mail.ioa.ac.cn (J.Z.); daiyuyu@mail.ioa.ac.cn (Y.D.); 3 Beijing Engineering Research Center of Sea Deep Drilling and Exploration, Institute of Acoustics, Chinese Academy of Sciences, Beijing 100190, China

**Keywords:** multi-objective optimization, cylindrical ultrasonic array, NSGA-II algorithm, BPNN

## Abstract

Key detection performance metrics, particularly resolution, are largely determined by the design parameters of ultrasonic arrays. The structural design of the transducer strongly influences critical indicators, including side lobe levels, beam directivity, and focal spot size. To improve parameter selection, this study proposes a multi-objective optimization strategy specifically tailored for cylindrical surface ultrasonic transducers. The geometric parameters of the array and the variables influencing resolution performance are mapped in a nonlinear manner. The NSGA-II algorithm is employed to perform extremum seeking optimization on a trained BPNN, generating a Pareto-optimal solution set by specifying main-lobe width, side-lobe intensity, and sound-pressure uniformity as optimization objectives. For validation, the geometric configurations derived from this solution set are applied in acoustic field simulations. Simulation results demonstrate that the dynamic aperture exhibits clear regularity when the array settings meet millimeter-level resolution requirements. These findings support real-world engineering applications and provide valuable insights for enhancing the geometric design of cylindrical ultrasonic arrays.

## 1. Introduction

Oil casing is an essential component in oilfield drilling and production operations, employed to reinforce the wellbore and protect the structural integrity of the oil well. It plays a critical role in maintaining production stability and ensuring drilling safety. However, the subterranean environment is characterized by extremely corrosive fluids, as well as sustained high temperature and pressure [1]. These extreme conditions directly cause damage to oil well casings. Under moderate conditions, local corrosion and deformation occur, whereas under severe conditions, fatal defects such as perforations, fractures, and even disconnections may develop. Such failures can directly interrupt normal oil well production and may even trigger severe safety hazards, including formation fluid leakage and blowouts [2]. Therefore, the development of high-performance casing corrosion detection instruments and technologies is urgently required.

This urgency is further underscored by the fact that, in recent years, the frequency of wellbore damage in casing wells has increased annually as a result of extended oilfield development and greater exploitation depth [3,4]. In this context, the development of higher-performance casing detection tools and more effective detection technologies is essential to enable the identification of minor corrosion and damage, provide timely corrosion warnings, and facilitate early targeted interventions. Such advancements hold substantial practical value in enhancing oilfield development efficiency and reducing operation and maintenance costs, while also providing strong assurance of safe oil well operations.

Widely used detection techniques for pipeline interiors, such as magnetic flux leakage, radiography, ultrasonics, and electromagnetic methods [5], generally suffer from low detection resolution or are unsuitable for high-temperature and high-pressure subterranean conditions. Moreover, these methods are not well-suited to the specific requirements of oil casing wall detection. Ultrasonic imaging detection technology was the first logging method capable of generating clear images of the wellbore wall [6]. However, in conventional ultrasonic wellbore imagers, the detection resolution is constrained by the operating frequency of the transducer, typically achieving only centimeter-level accuracy. Furthermore, these systems struggle to adapt to complex wellbore diameters with substantial variations, further underscoring their limitations.

The annular array transducer has attracted considerable attention in multi-domain detection technologies due to two principal advantages: (1) it enables blind-spot-free detection through its 360° omnidirectional field-of-view coverage; and (2) it simultaneously achieves greater detection depth and higher resolution at the same operating frequency as conventional array structures. Significant advancements have also been realized in the fields of nondestructive testing and medical ultrasonography [7].

In the field of medical ultrasound, Moini et al. successfully developed a forward-looking annular array endoscopic device [8]. Using a delay-control algorithm, the cylindrical annular array transducer proposed by Yang et al. [9] can flexibly realize dynamic focusing and precise steering of the axial acoustic beam, thereby expanding the application range of annular arrays in medical ultrasound detection.

**Figure 1:**
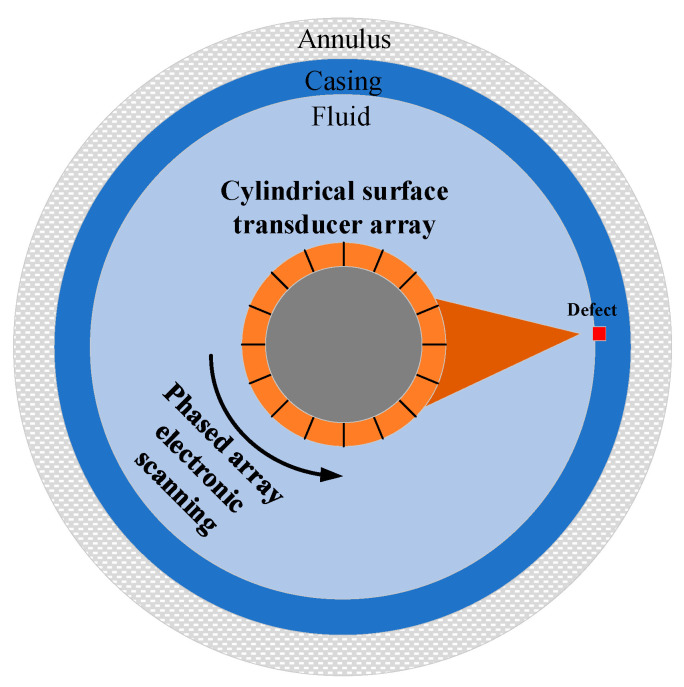
Working profile of a phased array on cylindrical surfaces in casing wells.

In the field of nondestructive testing, annular array transducers play a crucial role in detecting complex structures. Vos et al. performed a ground wellbore imaging experiment with a 128-element ultrasonic cylindrical linear phased array transducer operating at a center frequency of 1 MHz, thereby verifying the detection capability of multi-element circular arrays [10]. Zhang et al. researched phased array wellbore imaging using a self-constructed ground-based phased array transmission and acquisition system [11]. These studies further advance the integration of cylindrical annular array transducers with ultrasonic phased-array technology, offering a new pathway toward more precise and efficient nondestructive testing. The operating mechanism of the cylindrical annular array transducer within the well is illustrated in Figure 1.

Compared with single transducers, the various array transducers described above offer significant advantages in detection coverage, sensitivity, and imaging resolution through optimized geometric design, detection devices, and methodologies. Early studies on array parameter optimization primarily concentrated on single-parameter approaches. Dolph et al. proposed an analytical optimization scheme that employed the minimization of the main lobe width of the acoustic beam as the objective function, deriving and optimizing the weighting function of the variable aperture in a one-dimensional linear array. By adjusting the aperture weighting distribution, efficient concentration of main lobe energy was achieved [12]. Wooh and Shi, in contrast, adopted minimization of the side lobe level as their core objective. For linear arrays, they derived the optimal theoretical solutions for array element width and emission aperture, thereby providing a clear design basis for reducing side lobe interference [13]. Although single-parameter optimization can efficiently yield optimal solutions through analytical methods, it neglects the coupling effects among array parameters and thus fails to achieve coordinated improvement across multiple performance indicators. With the growing complexity of array structures and the demand for synergistic enhancement of multiple performance metrics, the limitations of single-parameter optimization have become increasingly evident. Against this backdrop, multi-parameter optimization techniques based on global optimization methods have emerged as the mainstream approach. Blum et al. addressed the challenges of energy focusing and dispersion in spherical arrays by employing genetic algorithms to simultaneously optimize six core array parameters [14]. Li et al. developed a multi-objective optimization model for the comprehensive performance enhancement of linear arrays, integrating both directivity and far-field deflection direction into a unified framework to achieve coordinated improvement of these two key indicators [15].

However, existing studies on multi-parameter optimization still exhibit notable limitations: (1) most optimization efforts have focused primarily on the geometric parameters of linear arrays, while systematic optimization studies on the geometric parameters of cylindrical arrays remain scarce, making it difficult to meet the application requirements of cylindrical arrays in specialized scenarios such as wellbore detection; and (2) the existing optimization results are largely limited to multiple discrete solutions within a prescribed parameter range, without in-depth exploration of the inherent principles and correlation mechanisms underlying geometric parameter design. As a result, the optimization models exhibit weak generalization ability and cannot be effectively transferred to array design scenarios with varying specifications or diverse detection requirements.

The hybrid combination of Backpropagation Neural Network (BPNN) and Genetic Algorithm (GA)-based methods has been widely documented in the literature. Ding et al. proposed a GA-based optimization of BPNN to accelerate convergence and improve generalization [16]. Venkatesan et al. applied a GA–Artificial Neural Network (ANN) model in manufacturing process optimization and reported time savings and improved accuracy [17].

Although the hybrid method combining and BPNN has been successfully applied in numerous fields, its application to the geometric optimization of cylindrical ultrasonic phased array transducers remains unexplored. Against this backdrop, this paper introduces the BPNN-Non-dominated Sorting Genetic Algorithm II (NSGA-II) framework for the first time to systematically address this optimization challenge.

## 2. Optimization Parameters and Objective Function

The primary parameters characterizing the imaging performance of ultrasonic testing include imaging resolution, detection depth, signal-to-noise ratio (SNR), and spatial positioning accuracy. These parameters are primarily coupled and influenced by factors such as ultrasonic transducer characteristics (number of dynamic array elements, element spacing, and center frequency), detection system parameters (transmission power, pulse width, and sampling frequency), and detection process parameters (probe incident angle, coupling medium type and thickness, and scanning step size). Given that the simulation stage is dominated by ultrasonic transducers, their parameters represent the primary factors influencing imaging performance. In this study, the center frequency of the ultrasonic transducer, the number of dynamic array elements, and the element spacing are selected as the primary input parameters for optimization. The optimization objectives are the main lobe width, side lobe level, and sound pressure uniformity, which collectively characterize the imaging performance of ultrasonic corrosion detection.

### 2.1. Acoustic Field of the Cylindrical Phased Array

In transducer array design, array directivity is one of the core indicators constraining overall system performance. The spatial distribution characteristics of directivity directly determine the accuracy with which the transducer controls the acoustic field and its adaptability to different applications. The number of dynamic array elements, the center frequency, and the element spacing, which are core parameters of array design, can be adjusted by controlling the spatial interference superposition of acoustic waves as described by the directivity function. In terms of the three key metrics—main lobe width, side lobe level, and sound pressure uniformity, these parameters collectively govern the acoustic field characteristics of the transducer array, thereby influencing the system’s effective resolution in target detection scenarios.

In this study, acoustic simulations were performed assuming a homogeneous aqueous medium with a sound velocity of 1500 m/s and a density of 1000 kg/m^3^, which are consistent with the acoustic properties of coupling fluids typically used in wellbore inspections. To focus on the geometric optimization of the transducer array, acoustic attenuation and electromechanical coupling effects were neglected. It should be noted that in practical applications, coupling materials, whose acoustic impedance is very close to that of water, exhibit similar acoustic behaviors. Therefore, their influence on the acoustic field distribution is minimal, and excluding attenuation and coupling effects allows the analysis to concentrate on the primary objective of this study—the geometric optimization of the transducer array.

The total radiated sound pressure of the cylindrical phased array is expressed in Equation (1) [18]:


(1) $$p(r,\theta ,z)=\sum_{j=1}^{2N_{\mathrm{total}}+1}p_{j}e^{-i\Delta \phi_{j}}$$

In the Equation, a cylindrical coordinate system is adopted, with the origin located at the center of the cylinder. Here, r denotes the radial coordinate, θ the tangential coordinate, and the z axial coordinate. $N_{\mathrm{total}}$ represents the number of transducer elements in the cylindrical array, $p_{j}$ represents the radiated sound pressure of the j-th element at the spatial field point (r,θ,z), and $\Delta \phi_{j}$ denotes the phase delay of the j-th element at the same field point.

The directivity function D(θ,z) is defined as the ratio of the sound pressure amplitude in a given spatial direction to the maximum sound pressure amplitude at the focal point, and is used to quantify the spatial directivity characteristics of the array acoustic beam. The directivity function of the cylindrical phased array can be derived from Equation (1):

(2) $$D(\theta ,z)=\frac{\left|p(r,\theta ,z)\right|}{P\max}=\frac{|\sum_{j=1}^{2N_{\mathrm{total}}+1}A_{j}e^{i(\Delta \phi_{j}-\Delta \phi_{j0})}|}{\sum_{j=1}^{2N_{\mathrm{total}}+1}A_{j}}$$

In the equation, $A_{j}=p_{j}e^{ik(r_{j}-r_{0})}/r_{j}$ represents its complex acoustic pressure contribution at the observation point after propagation and phase compensation. Furthermore, owing to the symmetry of the cylindrical array, no phase variation occurs along the z axial direction, while the tangential direction θ serves as the principal scanning direction. Accordingly, the directivity function can be simplified to a form dependent solely on the tangential direction θ.


(3) $$D(\theta )=\frac{|\sum_{j=1}^{2N_{\mathrm{total}}+1}A_{j}e^{ikR_{0}[\cos(\theta -\theta_{j})-\cos(\theta_{0}-\theta_{j})]}|}{\sum_{j=1}^{2N_{\mathrm{total}}+1}A_{j}}$$

In the equation, k denotes the wavenumber, $R_{0}$ the cylinder radius, $\theta_{0}$ the tangential angle of the focal point, and $\theta_{j}$ the tangential angle of the j-th element.

### 2.2. Performance Parameters of the Cylindrical Phased Array

#### 2.2.1. Main Lobe Width

The main lobe width, also referred to as the Half Power Beam Width (HPBW), is defined as the angular range over which the directivity function decreases from its peak value to −3 dB, and is expressed by the following equation:


(4) $$\mathrm{HPBW}=\theta_{high}-\theta_{low}$$

In the equation, $\theta_{high}$ denotes the maximum angle at which the main lobe peak decreases to −3 dB, while $\theta_{low}$ denotes the minimum angle at which the main lobe peak decreases to −3 dB.

#### 2.2.2. Side Lobe Level

The side lobe level (SLL), also referred to as the Peak Side Lobe Level (PSLL), is defined as the decibel ratio of the maximum side lobe amplitude to the peak amplitude of the main lobe. It can be expressed by the following equation:


(5) $${\mathrm{PSLL}=20\log}_{10}(\frac{\max(D(\theta )|\theta \notin [\theta_{low,}\theta_{high}])}{\max(D(\theta ))})$$

#### 2.2.3. Sound Pressure Distribution Uniformity

**Figure 2:**
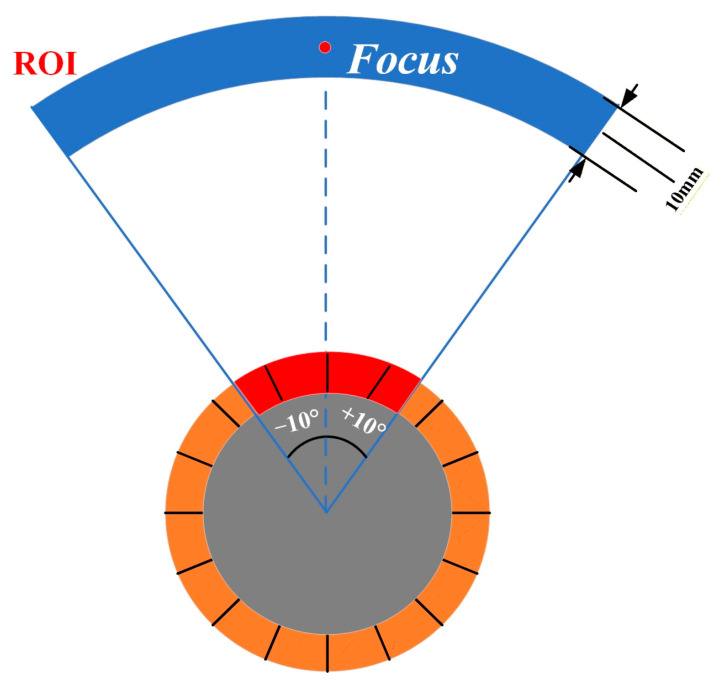
Schematic illustration of the fan-shaped ROI.

The sound pressure distribution uniformity (SPDUNI) is quantified as the standard deviation of the normalized sound-pressure amplitude within a defined Region of Interest (ROI). The ROI covers an angular range of ±10° and extends axially 5 mm before and 5 mm after the focal position, forming a fan-shaped zone around the focus for evaluating near-focal uniformity, which is represented in Figure 2. The equation is expressed below:


(6) $$\mathrm{SPDUNI}=\sqrt{\frac{1}{M-1}\sum_{m=1}^{M}{\left(p(\theta_{m})-\bar{p}\right)}^{2}}$$

In the equation, M denotes the number of sampling angles within the ROI, $\theta_{m}$ the m-th sampling angle in the ROI, $p(\theta_{m})$ the sound pressure amplitude at angle $\theta_{m}$ and $\bar{p}$ represents the mean amplitude of all sampled sound pressures within the ROI.

### 2.3. Multi-Objective Function

The geometric parameters of the array form a coupled regulatory network through the interference superposition mechanism of the directivity function, collectively determining HPBW, PSLL, and SPDUNI. For the design of cylindrical ultrasonic arrays, a multi-objective optimization function model must be constructed based on the high-resolution performance requirements of borehole detection, enabling global optimization of acoustic field characteristics through a multi-dimensional parameter collaborative optimization algorithm.

Taking HPBW, PSLL, and SPDUNI as the core objectives, the following multi-objective function is formulated:


(7) $$\mathrm{Score}(x)=w\cdot {\bar{y}}_{i}(x)$$

In the equation, Score(x) denotes the scalar score function, w the weight vector, ${\bar{y}}_{i}(x)$ the normalized form of the objective function, where i characterizes HPBW, PSLL, and SPDUNI.


(8) $${\bar{y}}_{i}(x)=\frac{y_{i}(x)-y_{i\_\min}(x)}{y_{i\_\max}(x)-y_{i\_\min}(x)}$$

The optimization variable vector x consists of the geometric parameters of the array:


(9) $$x=[N,f_{c},d]$$

In the equation, N denotes the number of dynamic array elements, $f_{c}$ the center frequency, and d the array element spacing.

The Pareto solution sets are ranked according to the weighted sum formula. Through this ranking process, the geometric parameter solutions with the best overall performance are identified.

### 2.4. Constraints

In petroleum engineering, geological exploration, and wellbore maintenance, accurate detection of inner-wall defects in casing wells—as a core component of the wellbore structure—directly determines the safe service life of the well and the stability of mining operations [19]. Cylindrical ultrasonic phased array systems have emerged as a core technology for borehole defect detection owing to their dynamically adjustable beam directivity, high imaging resolution, and wide coverage capability [20]. However, due to inherent size limitations of cased wells, the diameter of the cylindrical transducer is constrained to a specific value. A diameter of 76 mm is adopted in accordance with the parameter design of most downhole transducers, which not only accommodates the majority of small- and medium-sized cased wells, but also ensures adequate structural strength of the transducer. Nevertheless, this fixed constraint affects the interdependent relationship among core parameters—namely, element number, element spacing, and operating frequency—of the ultrasonic phased array system. Therefore, the following physical constraints are introduced to ensure accurate alignment with practical engineering conditions and to provide a reliable basis for simulation.

- Dynamic array element number N: 4≤N≤64, The value of N should ensure sufficient aperture and beam control accuracy while avoiding excessive hardware complexity and increased cost.
- Center frequency $f_{c}$: $500\;\mathrm{kHz}\leq f_{c}\leq 1\;\mathrm{MHz}$, The selected value of $f_{c}$ must balance the penetration capability of the acoustic wave in the wellbore medium with the ability to resolve defects.
- Array element spacing d: The spacing d, associated with the deflection angle θ, $0^{{}^{\circ}}\leq \theta \leq 90^{{}^{\circ}}$ of the acoustic beam, must be constrained to prevent the generation of grating lobes.

(10) $$\begin{array}{l} N_{\min}\leq N\leq N_{\max},\\ f_{c,\min}\leq f_{c}\leq f_{c,\max},\\ d\leq \frac{\lambda }{1+|\sin\theta |}. \end{array}$$

## 3. Optimization Methods

**Figure 3:**
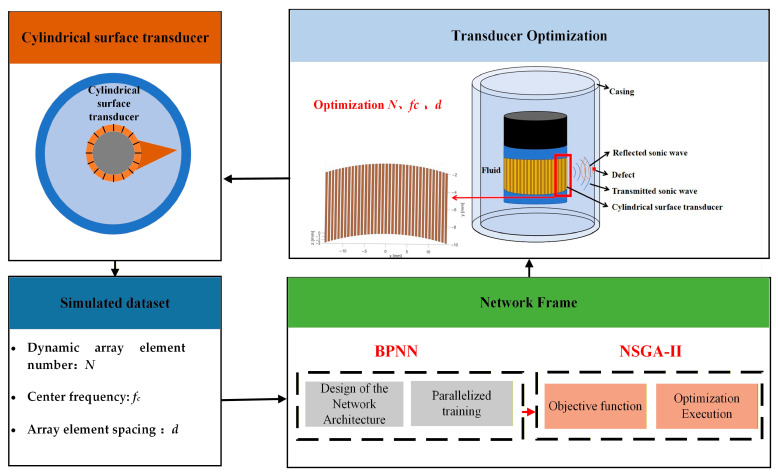
Overall optimization scheme flowchart.

The optimization approach primarily employs the NSGA-II in combination with BPNN. The flowchart of the overall optimization scheme is presented in Figure 3.

### 3.1. BPNN

**Figure 4:**
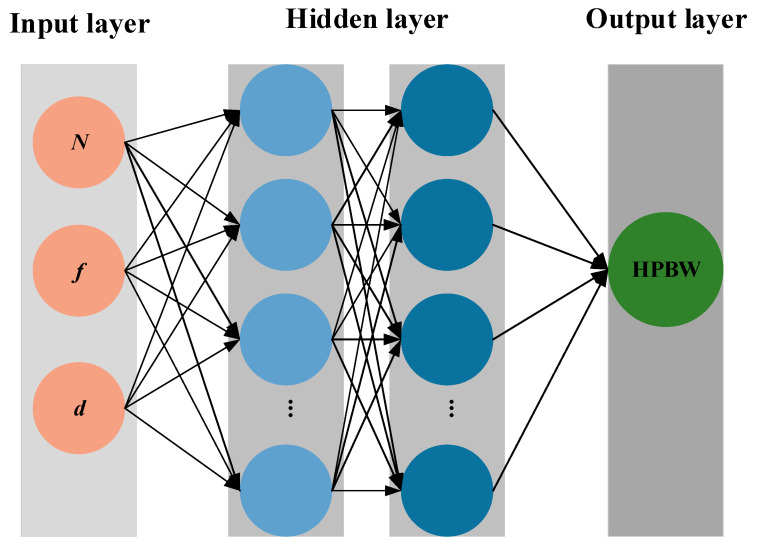
Multi-layer network mapping of single-objective optimization HPBW.

As a multilayer feedforward neural network employing forward propagation for output generation and backpropagation for weight adjustment, the BPNN can effectively model complex nonlinear mapping relationships through iterative optimization [21] and has demonstrated significant application value in optimizing ultrasonic phased array acoustic field parameters [22,23]. This approach effectively captures the implicit coupling relationships between geometric parameters and acoustic field performance indicators, while overcoming the limitations of traditional analytical methods that rely on numerous simplifying assumptions and have restricted applicability. The working principle of the BPNN is illustrated in Figure 4. Compared with conventional methods, the proposed approach improves prediction and optimization efficiency by one to three orders of magnitude, and exhibits strong robustness to interference and noise, making it suitable for real-time engineering design scenarios.

However, in multi-objective optimization, the BPNN often requires either aggregating multiple objectives into a single composite objective or constructing separate models for each objective, making it difficult to achieve independent optimization of multiple objectives simultaneously. Moreover, its performance is highly dependent on the availability of large quantities of high-quality samples, and the absence of universal criteria for hyperparameter selection increases the risk of both overfitting and underfitting. To overcome the shortcomings of the BPNN in multi-objective processing, data dependence, and hyperparameter sensitivity, this study employs the NSGA-II algorithm to address the limitations of the BPNN in multi-objective optimization.

### 3.2. NSGA-II

**Figure 5:**
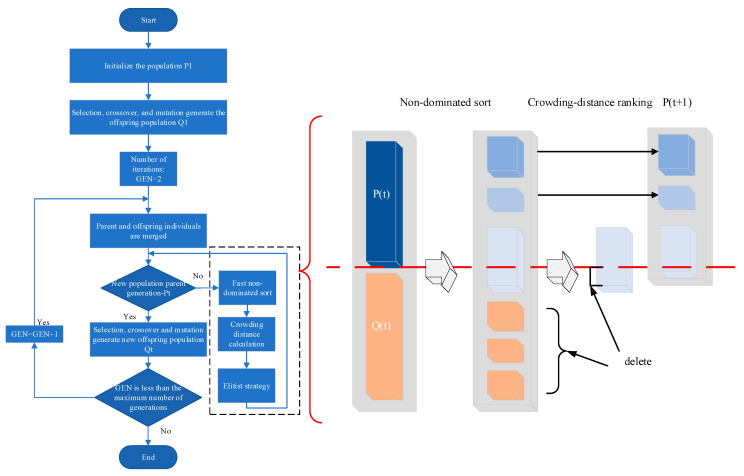
Working flowchart of the NSGA-II algorithm.

The NSGA-II algorithm is a multi-objective evolutionary optimization method built upon the genetic algorithm framework. Its core mechanisms include fast non-dominated sorting, crowding distance calculation, and an elitist retention strategy, which enable efficient exploration of the solution space and the generation of a uniformly distributed set of Pareto-optimal solutions without aggregating multiple objectives into a single one [24]. The workflow of NSGA-II is illustrated in Figure 5.

In the optimization design of cylindrical ultrasonic phased arrays, the NSGA-II algorithm can simultaneously address multiple competing objectives without relying on weighted aggregation, directly producing a set of Pareto-optimal solutions to meet diverse imaging requirements. This capability effectively overcomes the inherent limitations of traditional methods, such as BPNN, in handling multi-objective conflicts. More importantly, NSGA-II can directly incorporate physical constraints, such as the no-grating-lobe condition, into the evolutionary process, thereby ensuring the feasibility and engineering applicability of the solutions from the outset of the search. In addition, the global optimization mechanism based on population evolution markedly reduces the dependence on large amounts of labeled data required by BPNN, while avoiding underfitting and overfitting problems caused by insufficient training samples or distribution deviations. Consequently, it provides a more efficient, robust, and flexible solution for the collaborative optimization of the geometric and acoustic performance of cylindrical ultrasonic phased arrays.

## 4. Simulation of the Optimization Results

### 4.1. Single-Objective Optimization Using BPNN

#### 4.1.1. Construction of BPNN

First, a modeling and optimization approach based on the BPNN was employed to achieve effective control of the main-lobe beamwidth (HPBW). A simulated dataset was generated, in which three design parameters—the dynamic array element number, center frequency, and element spacing—served as inputs, while the beamwidth acted as the single output variable. The dataset contained 2000 samples, with all input parameters randomly distributed within the specified physical constraints. Before training, the input data were standardized and normalized to the range [0, 1] to enhance the stability and convergence efficiency of the neural network.

A BPNN with two hidden layers (containing 16 and 8 neurons, respectively) was constructed and trained using the Levenberg–Marquardt algorithm [25]. The dataset was divided into training, validation, and test sets in a ratio of 7:1.5:1.5. The network employed the Mean Squared Error (MSE) as the performance function, with the maximum number of training epochs set to 1000. After training, the predictive performance for HPBW was evaluated on the test set, and the MSE, Mean Absolute Error (MAE), and coefficient of determination (R^2^) were calculated as quantitative metrics.

#### 4.1.2. Performance of BPNN

**Figure 6:**
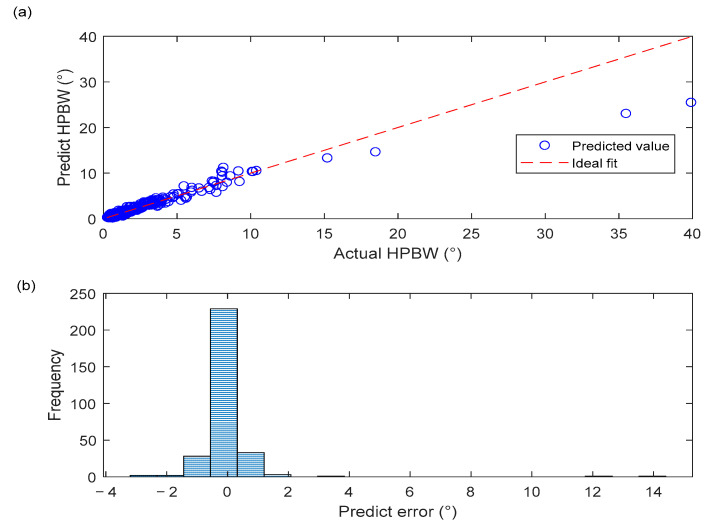
(**a**) Prediction performance of HPBW; (**b**) Distribution of HPBW prediction errors.

The BPNN model developed in this study exhibits strong predictive performance. As shown in Figure 6a, the scatter points representing the predicted values are closely clustered around the ideal fitting line, indicating high consistency between the predicted and actual HPBW values. In particular, in the 0–5° region where HPBW values are small, the predicted points almost entirely coincide with the fitting line, clearly demonstrating the high predictive accuracy of the model within this interval. It is noteworthy that in regions where HPBW exceeds 5°, although some data points exhibit slight deviations, the overall trend remains linear, reflecting the good generalization capability of the model. Figure 6b, which illustrates the distribution of HPBW prediction errors, further shows that the vast majority of errors are concentrated near zero, with an approximately normal distribution and no significant bias. This strongly demonstrates that the training process was both stable and effective.

**Figure 7:**
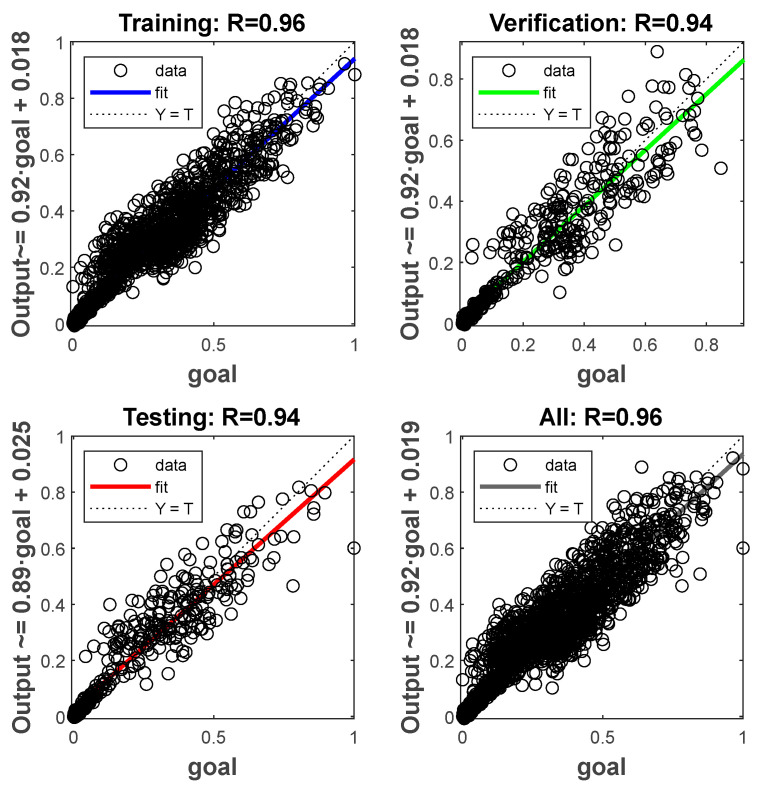
Regression performance of the BPNN model.

Figure 7 presents the regression performance of the BPNN model. For the training set, the correlation coefficient is 0.96, with data points closely distributed along the ideal Y = T line, indicating that the model accurately captures the complex nonlinear mapping between inputs and outputs in the training samples. For both the validation and test sets, the correlation coefficient is 0.94. The data points of both sets are closely clustered around the ideal fitting line, with only slight deviations observed in the high-value region of the test set, reflecting the model’s strong generalization capability and absence of overfitting. For all data combined, the correlation coefficient is 0.96, with data points closely distributed around the fitting line, further verifying the high linear correlation between the model’s predictions and the actual target values. These results confirm the reliability and accuracy of the neural network constructed as a surrogate model for acoustic field performance, thereby laying a solid foundation for the subsequent parameter optimization stage.

#### 4.1.3. Single-Objective Optimization Results

**Figure 8:**
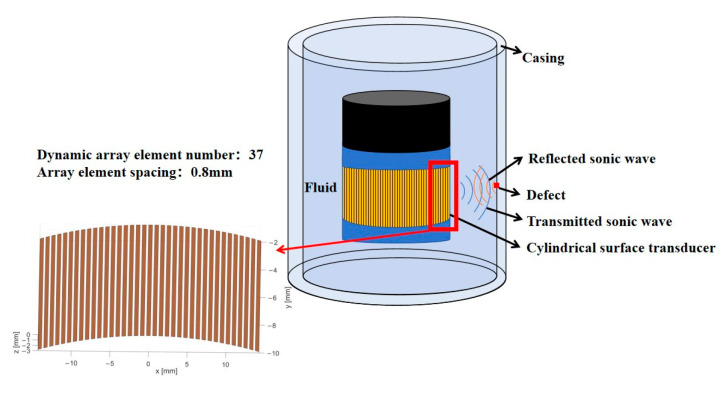
Dynamic aperture shape of the cylindrical transducer corresponding to the optimized BPNN geometric parameter solution.

During the parameter optimization stage, a global search was conducted to minimize HPBW while ensuring that all constraints were satisfied. The optimization results indicate that the optimal combination of geometric parameters includes 37 dynamic sensors, a center frequency of 722 kHz, and an array spacing of 0.80 mm. Under these conditions, the HPBW reaches a minimum value of 2.99°, with all constraints satisfied. Figure 8 presents a schematic diagram of the dynamic aperture shape of the cylindrical transducer corresponding to the optimized geometric parameters.

**Figure 9:**
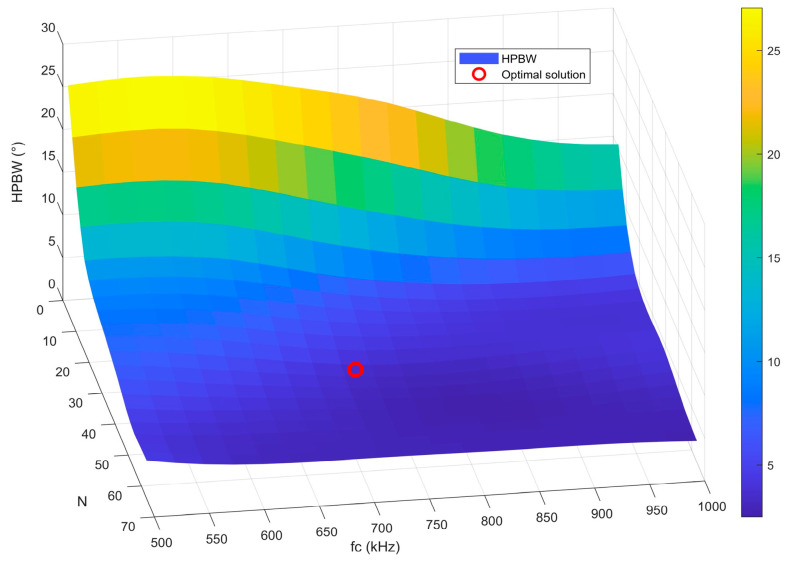
Response characteristics of HPBW as a function of the number of array elements and the center frequency.

Furthermore, the response characteristics of HPBW with respect to variations in the number of array elements and center frequency, under the condition of optimal element spacing, are illustrated by the three-dimensional surface in Figure 9. This visualization intuitively reveals the coupling relationships among parameters and highlights the location of the optimal solution. As shown in the figure, under optimal spacing, when the number of array elements is fixed, higher frequencies correspond to narrower HPBW. When the frequency is fixed, increasing the number of array elements results in a narrower HPBW. Moreover, the influence of the number of array elements on HPBW is particularly significant. Overall, as the number of array elements increases, the HPBW shows a clear decreasing trend, with the effect of array elements becoming more prominent.

Moreover, when PSLL and SPDUNI were considered as independent optimization objectives, the resulting optimal parameter combinations differed. When PSLL was selected as the target, the optimal solution corresponded to 27 dynamic array elements, a center frequency of 722 kHz, and an element spacing of 1.00 mm. When SPDUNI was selected as the target, the optimal solution corresponded to 17 dynamic sensors, a center frequency of 555.6 kHz, and an element spacing of 0.80 mm. Further analysis revealed that the solutions generated by BPNN consistently exhibited variations when the optimization objective was changed. This indicates that BPNN cannot simultaneously address multi-objective optimization problems, making it difficult to balance the requirements of HPBW, PSLL, and SPDUNI within a single optimization. In practical applications, complex coupling relationships often exist among these indicators, and focusing solely on a single objective may result in poor performance in other aspects, thereby preventing the achievement of optimal overall performance.

### 4.2. Multi-Objective Optimization Using NSGA-II

#### 4.2.1. Construction of NSGA-II

To overcome the limitation that the single-objective BPNN optimization can only target one performance metric—typically HPBW—while neglecting interdependencies among other key indicators such as PSLL and SPDUNI, this study develops a hybrid multi-objective optimization framework that integrates NSGA-II with BPNN to achieve balanced optimization of multiple acoustic-field parameters. This framework uses HPBW as the primary optimization objective and incorporates PSLL and SPDUNI, thereby forming an optimization paradigm centered on a three-dimensional objective space.

Each chromosome in the NSGA-II framework encodes a potential design solution consisting of three decision variables—the number of dynamic array elements (N), the center frequency ($f_{c}$), and the element spacing (d). These variables collectively determine the phased array’s acoustic-field performance through their effects on HPBW, PSLL, and SPDUNI.

With respect to algorithm parameter configuration, a population size of 100 provides sufficient genetic diversity, ensuring comprehensive exploration of the three-dimensional design variable space (N, $f_{c}$, and d) as well as the three-dimensional objective space (HPBW, PSLL, and SPDUNI). Setting the number of generations to 500 ensures adequate inheritance, recombination, and optimization of favorable genes, allowing the algorithm to gradually converge toward a uniformly distributed and widely covered Pareto optimal solution set.

The successful construction of this multi-objective optimization framework enables the collaborative optimization of the three conflicting performance metrics—HPBW, PSLL, and SPDUNI, building upon the high-precision HPBW prediction achieved by the single-objective BPNN.

#### 4.2.2. Multi-Objective Optimization Results

**Figure 10:**
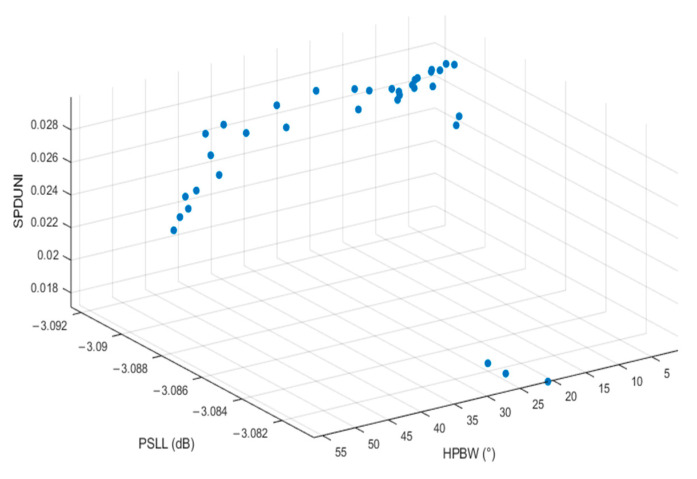
Pareto optimal solution set in the three-dimensional objective space (HPBW, PSLL, and SPDUNI).

The trained BPNN algorithm accurately predicts the output parameters HPBW, PSLL, and SPDUNI from the three input variables: number of dynamic sensors, center frequency, and sensor spacing. The set of optimal solutions obtained using the NSGA-II algorithm is illustrated in Figure 10, where each blue circle represents a Pareto optimal solution corresponding to a different trade-off among HPBW, PSLL, and SPDUNI.

As shown in the three-dimensional Pareto front distribution, the solution set contains a large number of non-dominated solutions, each exhibiting distinct dominant characteristics across different performance indicators. When HPBW in the solution set is very small, the focusing ability is enhanced; however, PSLL and SPDUNI may not reach ideal values. This may result in artifact interference in the detection region and insufficient signal stability. Each solution has unique advantages within a specific performance dimension but is constrained by the physical limitations of probe design, making it difficult to directly determine a global optimal solution based solely on the solution set.

For the core requirement of “resolution first” in casing wall defect detection, where the ability to identify small and hidden defects must be prioritized, HPBW is the decisive factor for detection resolution. A smaller HPBW indicates stronger ultrasonic focusing and, consequently, greater capability to recognize small defects. Therefore, it is necessary to transform these qualitative requirements into a quantitative decision-making basis by assigning differentiated weight vectors. The optimization direction and quantitative standards of the three objectives are defined as follows:

- HPBW: Reflects signal focusing ability; the smaller the value, the better the performance. As the core objective, it has the highest priority, directly corresponding to the “resolution-first” requirement.
- PSLL: Reflects anti-interference capability; the smaller the value (i.e., the more negative), the better the performance. It serves as a secondary objective.
- SPDUNI: Reflects signal stability; the smaller the value, the better the performance. It serves as a secondary objective.

By assigning three objective weight vectors $w=[w_{1},w_{2},w_{3}]=[0.8,0.1,0.1]$, the optimization process ensures that the core requirement of small defect identification is prioritized.

The Pareto solution sets are ranked according to the weighted sum formula, with the three BPNN solution sets incorporated into the ranking system to compute comprehensive scores. Through this ranking process, the geometric parameter solutions with the best overall performance are identified. To further verify the engineering practicability and rationality of the selected parameter solution, the geometric parameters of the SPACE Vernier instrument, widely applied and recognized for its performance advantages in related fields, are introduced as a reference for comparative analysis [26]. Its configuration includes 32 dynamic array elements, a center frequency of 1 MHz, and an element spacing of 0.829 mm. In addition, Table 1 provides a detailed ranking of the solutions obtained using the weighted summation method and presents the corresponding scores for each parameter solution, thereby allowing a clear comparison of performance differences among the solutions.

**Figure 11:**
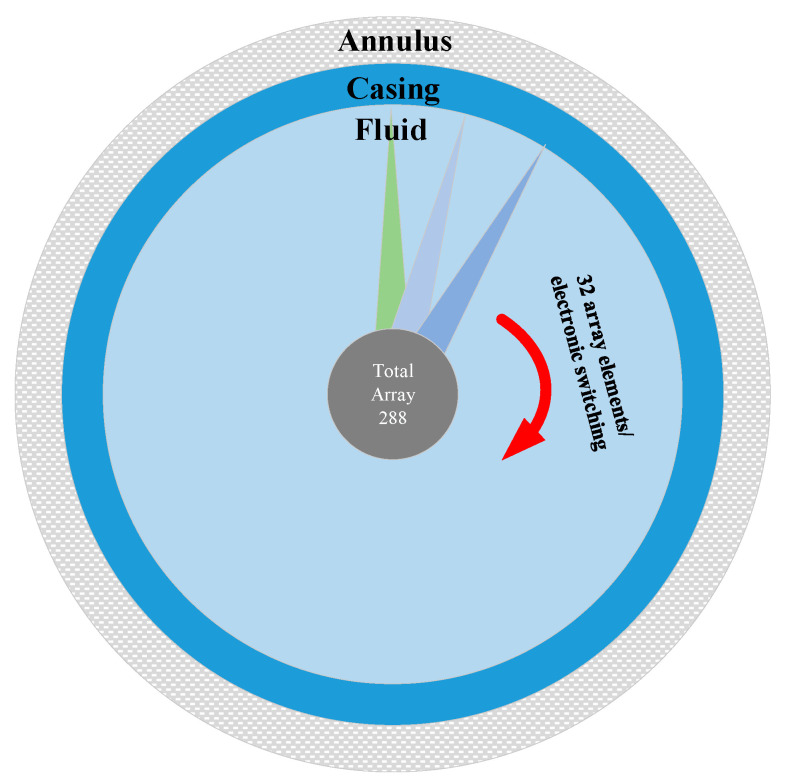
Working diagram of the SPACE Vernier instrument.

Additionally, Figure 11 presents the working diagram of the SPACE Vernier instrument within its casing, providing a clearer understanding of its geometrical structure and parameter design. This figure also provides solid theoretical support and an intuitive structural reference for subsequent engineering application analyses and discussions on parameter optimization.

## 5. Discussion

### 5.1. Acoustic Field Simulation

To verify the effectiveness of the geometric parameter solutions obtained through the single-objective BPNN optimization and the NSGA-II algorithm, this study employs the spatial impulse response method [27] to conduct acoustic field simulations. In this method, the focusing target of the cylindrical ultrasonic phased array is the inner wall of the casing, with the focal position located 90 mm from the transducer center. The pulse-echo response and the sound pressure distribution at arbitrary observation points in space are then calculated numerically based on the specified geometric parameter configuration.

Based on Huygens’ principle and linear acoustic theory, the spatial impulse response method constructs the array acoustic field distribution by superimposing the impulse responses generated by each element at the observation point. This method accurately characterizes diffraction effects and interference phenomena during ultrasonic propagation, making it suitable for evaluating focusing performance and acoustic field characteristics in complex structures. In this simulation, the −3 dB and −6 dB focal spot sizes in the focal region are calculated and analyzed to objectively evaluate the ability of different optimization algorithms to improve focusing accuracy.

#### 5.1.1. Acoustic Field Simulation Based on BPNN Optimized Parameters

**Figure 12:**
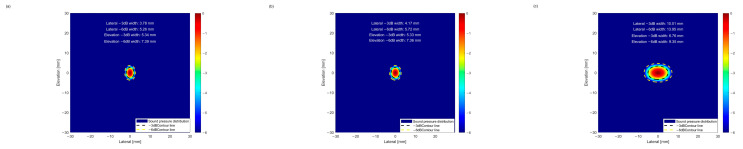
(**a**) Focusing of the BPNN1 beam; (**b**) Focusing of the BPNN2 beam; (**c**) Focusing of the BPNN3 beam.

First, acoustic field simulations were performed for the geometric parameter solutions obtained through single-objective BPNN optimization. The −3 dB point is generally regarded as the “half-power point,” where the sound energy is approximately half of the maximum value. The corresponding beam width directly reflects the focusing degree of the sound beam at the −3 dB energy level; smaller widths indicate better focusing performance and higher energy concentration. Lateral resolution is defined as one-quarter of the −6 dB beam width [28], representing the minimum separation between two adjacent defects at the same depth. This parameter reflects the minimum distinguishable distance between defects; smaller widths correspond to higher lateral resolution. As shown in Figure 12, the −3 dB and −6 dB values corresponding to the lateral and pitching dimensions of the three BPNN solutions reveal the focusing and resolving capabilities of the sound beam in the vertical direction, providing a comprehensive description of its spatial characteristics.

#### 5.1.2. Acoustic Field Simulation Based on NSGA-II Optimized Parameters

**Figure 13:**
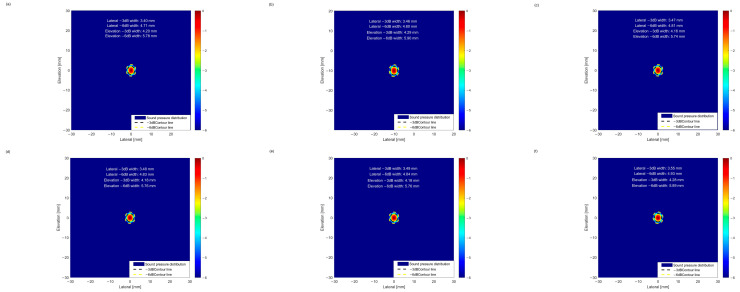
(**a**) Focusing of the NSGA-II 1 beam; (**b**) Focusing of the NSGA-II 2 beam; (**c**) Focusing of the NSGA-II 3 beam; (**d**) Focusing of the NSGA-II 4 beam; (**e**) Focusing of the NSGA-II 5 beam; (**f**) Focusing of the NSGA-II 6 beam.

Acoustic field simulations were performed for the geometric parameter results ranked by weighted summation in the NSGA-II algorithm, as shown in Figure 13.

**Figure 14:**
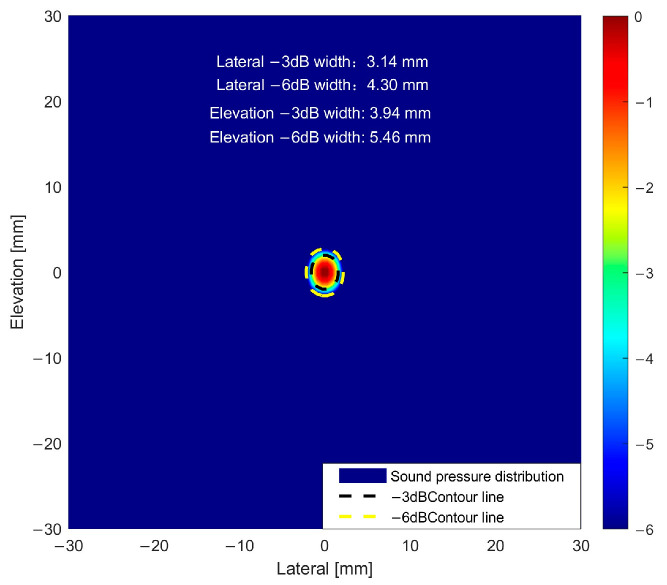
Focusing of the SPACE Vernier beam.

In order to systematically evaluate the ultrasonic focusing performance corresponding to different parameter solutions, this study quantized and compared the acoustic beam characteristics of the solutions obtained by the above BPNN and NSGA-II optimization algorithms and the acoustic beam characteristics of the actual parameter solutions of the SPACE Vernier instrument. The actual parameter focusing of the instrument is shown in Figure 14. The focus sharpness and energy concentration were characterized by the beam widths of −3 dB and −6 dB in the lateral and pitch directions, and the 10 sets of results are shown in Table 2.

From the ranking table of acoustic beam characteristics, it is evident that the four core indicators—lateral −3 dB width, lateral −6 dB width, elevation −3 dB width, and elevation −6 dB width, all follow the principle that smaller values indicate better performance. Within the optimization solution set, a clear gap exists between the BPNN and NSGA-II results, reflecting the limitations of single-objective BPNN optimization. The NSGA-II Solution 1 exhibits the best overall performance, with all four indicators ranked at the forefront, highlighting dual advantages in both lateral and elevation beam focusing. Solutions 2–6 of NSGA-II show a decline in performance, with the lateral −3 dB width exhibiting an increasing trend. Although the elevation indices are slightly better than those of Solution 1, the inferior lateral indices create a noticeable gap between these five solutions and the top-ranked solution. Nevertheless, the top six solutions collectively demonstrate comprehensive advantages far exceeding those of the BPNN set, particularly in combined lateral and elevation focusing performance, making them optimal choices for high-precision defect detection.

A quantitative comparison between the actual parameter configuration of the SPACE Vernier instrument and the optimization algorithm solutions reveals that the instrument exhibits clear hardware design advantages in lateral beam focusing, with its −3 dB and −6 dB widths outperforming all other solutions. It should also be noted that the acoustic beam characteristics of the NSGA-II multi-objective optimization solutions closely approximate those of the instrument. The lateral −3 dB width is concentrated in the range of 3.40–3.55 mm, and the lateral −6 dB width is in the range of 4.71–4.93 mm, slightly wider than the instrument but overall stable and comparable to its performance. This strongly demonstrates the effectiveness of the NSGA-II multi-objective optimization solution set, which approaches the beam focusing performance of mature instruments. In addition, it provides multiple sets of high-performance and reliable parameter alternatives for practical engineering scenarios, offering an important reference for the geometric design and optimization of cylindrical phased arrays.

### 5.2. Parameter Trends

**Figure 15:**
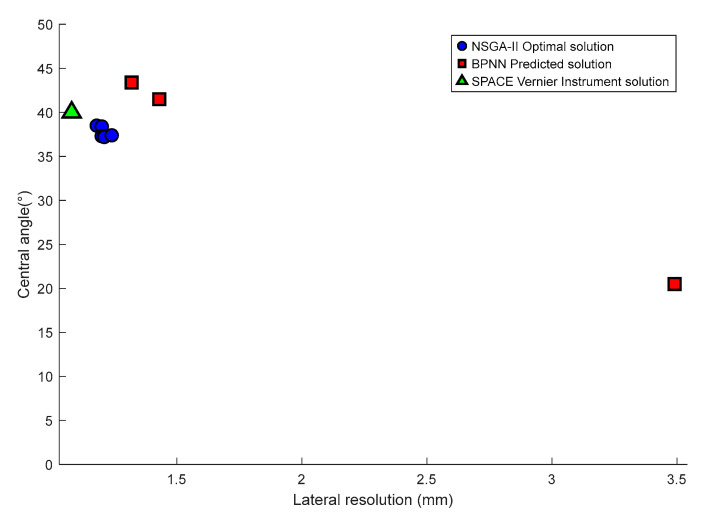
Central angle trends of BPNN predicted solutions, NSGA-II optimal solutions, and SPACE Vernier instrument solution dominated by lateral resolution.

Based on a comprehensive comparison of the single-objective optimization solutions obtained by BPNN, the multi-objective solution set generated by the NSGA-II algorithm, and the actual parameter configuration of the SPACE Vernier instrument, together with the application requirements for high-resolution circumferential inspection of casing wall defects. It is evident that when lateral resolution is prioritized, the top six NSGA-II solutions are more consistent with the optimization objectives than the other solution sets. Therefore, in analyzing the geometric parameter selection of the above ten solution sets, it is observed that a complex and strong coupling relationship exists between the array’s geometric parameters and the dynamic aperture size. As summarized in Figure 15 and the related analysis, the array’s geometric parameters and the central angle of the cylindrical surface corresponding to the dynamic aperture can be derived when lateral resolution is taken as the primary evaluation criterion.

From Figure 14 and the related analysis, it can be summarized that when lateral resolution is used as the primary evaluation criterion, the array geometric parameters (dynamic array element number, element spacing, and center frequency) and the central angle of the cylindrical surface corresponding to the dynamic aperture exhibit the following characteristics:

- Advantages of NSGA-II: The solutions obtained by NSGA-II provide superior resolution compared with those of the BPNN algorithm, and their beam focusing performance is highly comparable to that of the instrument solution. This fully verifies the technical effectiveness of NSGA-II in optimizing beam geometric parameters.
- Law of the Central Angle: Based on the performance data of the BPNN predicted solutions, the NSGA-II multi-objective optimization solution set, and the actual instrument configuration, when the lateral resolution of the ten core solutions ranges from 1.08 to 1.43 mm, achieving an overall high-precision level of approximately 1 mm, the corresponding central angle of the cylindrical surface lies within 37.2–43.4°.

This study proposes a multi-objective optimization-based design method for cylindrical ultrasonic phased array transducers. The optimization results exhibit strong consistency with the key parameters of existing commercial instruments, which effectively validates the effectiveness and reliability of the proposed approach. For subsequent research, we will focus on advancing the following work: structural optimization and fabrication of transducers, integrated development of data acquisition systems, implementation of real-time imaging algorithms, and experimental testing with comprehensive performance validation.

## 6. Conclusions

To address the requirements of transducer optimization design for high-resolution cylindrical phased-array defect detection in casing wellbores, this study proposes an optimization method based on array geometric parameters. The design focuses on three core performance parameters and systematically compares the differences between single-objective and multi-objective optimization.

Simulation results demonstrate that when the optimized geometric parameters achieve a lateral resolution of 1 mm in the acoustic field, and the beam focusing performance of the solution set closely approximates that of the actual instrument, the central angle of the cylindrical surface corresponding to the dynamic aperture consistently falls within the range of 37.2–43.4°. These findings provide a quantitative reference for the geometric optimization of cylindrical ultrasonic arrays and establish a theoretical foundation for their engineering application in casing wall defect detection.

Further analysis of the optimization results indicates that single-objective function optimization often produces significant discrepancies in parameter solutions across different objectives, whereas only multi-objective collaborative optimization ensures that the comprehensive performance of geometric parameter solutions reaches the optimal level. Future research will focus on the adaptive optimization of array parameters and detection methods tailored to different sizes of cased wells, to achieve adaptive beam adjustment of cylindrical ultrasonic phased arrays in complex downhole environments. This will further enhance the intelligence of downhole detection systems and enable flexible adaptation to the practical requirements of diverse detection scenarios.

## Figures and Tables

**Table 1:** Ranking of parameter solutions based on weighted summation score.

Rank	*N*	*f_c_* (kHz)	*d* (mm)	Score
SPACE Vernier	32	1000	0.829	0.093
NSGA-II 1	32	908.8	0.798	0.118
NSGA-II 2	32	914.7	0.796	0.142
NSGA-II 3	31	908.9	0.799	0.165
NSGA-II 4	31	918.4	0.797	0.169
NSGA-II 5	31	918.6	0.795	0.174
NSGA-II 6	31	924.2	0.797	0.178
BPNN 1	37	722.2	0.800	0.285
BPNN 2	27	722.2	1.000	0.465
BPNN 3	17	555.6	0.800	0.854

**Table 2:** Acoustic beam characteristics of different parameter solutions.

Parametric Solutions	Lateral −3 dB Width	Lateral −6 dB Width	Elevation −3 dB Width	Elevation −6 dB Width
SPACE Vernier	3.14	4.30	3.94	5.46
NSGA-II 1	3.40	4.71	4.20	5.78
NSGA-II 2	3.46	4.80	4.29	5.90
NSGA-II 3	3.47	4.81	4.16	5.74
NSGA-II 4	3.48	4.83	4.18	5.76
NSGA-II 5	3.49	4.84	4.18	5.76
NSGA-II 6	3.55	4.93	4.28	5.89
BPNN 1	3.78	5.26	5.34	7.39
BPNN 2	4.17	5.72	5.33	7.36
BPNN 3	10.01	13.95	6.76	9.35

## Data Availability

Data underlying the results presented in this paper are not publicly available at this time but may be obtained from the authors upon reasonable request.

## Abbreviations

The following abbreviations are used in this manuscript:

NSGA-II	Non-dominated Sorting Genetic Algorithm II
BPNN	Backpropagation Neural Network
HPBW	Half Power Beam Width
PSLL	Peak Side Lobe Level
SPDUNI	Sound Pressure Distribution Uniformity

## References

[1] Luo M., Feng Y.C., Gui Y., Deng J.G., Han C. (2021). Development status and prospect of key technologies for high temperature and high pressure drilling. Pet. Sci. Bull..

[2] Ajayi T., Gupta I. (2019). A review of reactive transport modeling in Wellbore Integrity Problems. J. Pet. Sci. Eng..

[3] Luo L.C., Zhang H.Z., Zhang J.M., Li T.W., Qiu M.X. (2024). Situation and Development Trend of Chinese and Foreign Oil and Gas Exploration and Development. Pet. Sci. Technol. Forum.

[4] Mohammed A.I., Oyeneyin B., Atchison B., Njuguna J. (2019). Casing structural integrity and failure modes in a range of well types—A Review. J. Nat. Gas Sci. Eng..

[5] Iizuka Y., Awajiya Y. (2014). High sensitivity EMAT system using chirp pulse compression and its application to crater end detection in continuous casting. J. Phys. Conf. Ser..

[6] Zemanek J., Glenn E.E., Norton L.J., Caldwell R.L. (1970). Formation evaluation by inspection with the Borehole televiewer. Geophysics.

[7] Demirci U., Oralkan O., Johnson J.A., Ergun A.S., Karaman M., Khuri-Yakub B.T. Capacitive micromachined ultrasonic transducer arrays for medical imaging: Experimental results. Proceedings of the 2001 IEEE Ultrasonics Symposium. Proceedings. An International Symposium (Cat. No.01CH37263).

[8] Moini A., Nikoozadeh A., Oralkan O., Choe J.W., Sarioglu A.F., Stephens D.N. Volumetric intracardiac imaging using a fully integrated CMUT ring array: Recent developments. Proceedings of the 2011 IEEE International Ultrasonics Symposium.

[9] Yang X.B., Yin G.J., Tian Y., Guo J.Z. (2020). Generating an Adjustable Focused Field with an Annular Shape Using a Cylindrical Acoustic Transducer Array. IEEE Trans. Ultrason. Ferroelectr. Freq. Control.

[10] Vos H.C.L., Schepers R., Vogel J.A. An ultrasonic circular array transducer for pipeline and borehole inspection. Proceedings of the IEEE 1988 Ultrasonics Symposium Proceedings.

[11] Dong H., Shi F.F., Kong C., Zhang B.X. Design of an ultrasonic phased array transducer for imaging borehole wall. Proceedings of the 2015 Symposium on Piezoelectricity, Acoustic Waves, and Device Applications (SPAWDA).

[12] Dolph C.L. (1946). A Current Distribution for Broadside Arrays Which Optimizes the Relationship between Beam Width and Side-Lobe Level. Proc. IEEE.

[13] Wooh S.C., Shi Y.J., Thompson D.O., Chimenti D.E. (1998). Optimization of Ultrasonic Phased Arrays. Review of Progress in Quantitative Nondestructive Evaluation.

[14] Blum F., Jarzynski J., Jacobs L.J. (2004). A focused two-dimensional air-coupled ultrasonic array for non-contact generation. NDT E Int..

[15] Li S., Xu C.G., Xiao D.G., Zhou S.Y., Yang T.X. (2009). Multi-Objective Optimal Mathematical Model of Geometrical Parameters of Ultrasonic Phased Arrays Transducer. Nondestr. Test..

[16] Ding S.F., Su C.Y., Yu J.Z. (2011). An optimizing BP neural network algorithm based on genetic algorithm. Artif. Intell. Rev..

[17] Venkatesan D., Kannan K., Saravanan R. (2009). A genetic algorithm-based artificial neural network model for the optimization of machining processes. Neural Comput. Appl..

[18] Deng F.Q., Zhang B.X., Wang D., Song G.P. (2006). Radiation Acoustic Field of a Linear Phased Array on a Cylindrical Surface. Chin. Phys. Lett..

[19] Prabhakara P., Mielentz F., Stolpe H., Behrens M., Lay V., Niederleithinger E. (2022). Validation of Novel Ultrasonic Phased Array Borehole Probe by Using Simulation and Measurement. Sensors.

[20] Liu X., Li Y.L., Qin H.G., Peng C. (2025). High-Frequency 64-Element Ring-Annular Array Transducer: Development and Preclinical Validation for Intravascular Ultrasound Imaging. Biosensors.

[21] Rumelhart D.E., Hinton G.E., Williams R.J. (1986). Learning representations by back-propagating errors. Nature.

[22] Chen D.D., Wang L.W., Luo X.J., Fei C.L., Li D., Shan G.B., Yang Y.T. (2021). Recent Development and Perspectives of Optimization Design Methods for Piezoelectric Ultrasonic Transducers. Micromachines.

[23] Wang S., Wang X.W., You F.C., Li Y., Xiao H. (2023). A Real Time Method Based on Deep Learning for Reconstructing Holographic Acoustic Fields from Phased Transducer Arrays. Micromachines.

[24] Deb K., Pratap A., Agarwal S., Meyarivan T. (2002). A fast and elitist multiobjective genetic algorithm: NSGA-II. IEEE Trans. Evol. Comput..

[25] Fischer A., Izmailov A.F., Solodov M.V. (2024). The Levenberg–Marquardt method: An overview of modern convergence theories and more. Comput. Optim. Appl..

[26] Troup D. Phased Array Ultrasonic Tools Provide Precise Measurement of Geometrically Complex Components Such as Nipple Profiles Downhole, Confirming Safety Critical Dimensions. Proceedings of the SPE/ICoTA’19 Well Intervention Conference and Exhibition.

[27] Stepanishen P.R. (1970). Transient Radiation from Pistons in an Infinite Planar Baffle. J. Acoust. Soc. Am..

[28] Dube N. (2004). Introduction to Phased Array Ultrasonic Technology Applications: R/D Tech Guideline.

